# Isolation and identification of a novel porcine-related recombinant mammalian orthoreovirus type 3 strain from cattle in Guangxi Province, China

**DOI:** 10.3389/fmicb.2024.1419691

**Published:** 2024-07-22

**Authors:** Yuhang Luo, Yanglin Wang, Wenfei Tang, Cui Wang, Huanghao Liu, Xiaoling Wang, Jiang Xie, Jie Wang, Kang Ouyang, Ying Chen, Zuzhang Wei, Yifeng Qin, Yan Pan, Weijian Huang

**Affiliations:** ^1^Laboratory of Animal Infectious Diseases and Molecular Immunology, College of Animal Science and Technology, Guangxi University, Nanning, China; ^2^Guangxi Vocational University of Agriculture, Nanning, China; ^3^Guangxi Zhuang Autonomous Region Engineering Research Center of Veterinary Biologics, Nanning, China; ^4^Guangxi Key Laboratory of Animal Breeding, Disease Control and Prevention, Nanning, China; ^5^Liuzhou Center for Animal Disease Control and Prevention, Liuzhou, China

**Keywords:** mammalian orthoreoviruses, bovine, diarrhea, isolation, reassortment, pathogenesis and transmissibility

## Abstract

The Mammalian orthoreovirus (MRV) infects various mammals, including humans, and is linked to gastrointestinal, respiratory, and neurological diseases. A recent outbreak in Liuzhou, Guangxi, China, led to the isolation of a new MRV strain, GXLZ2301, from fecal samples. This strain replicates in multiple cell lines and forms lattice-like structures. Infected cells exhibit single-cell death and syncytia formation. The virus’s titers peaked at 10^7.2^ TCID_50_/0.1 mL in PK-15 and BHK cells, with the lowest at 10^3.88^ TCID50/0.1 mL in A549 cells. Electron microscopy showed no envelope with a diameter of about 70 nm. Genetic analysis revealed GXLZ2301 as a recombinant strain with gene segments from humans, cows, and pigs, similar to type 3 MRV strains from Italy (2015-2016). Pathogenicity tests indicated that while the bovine MRV strain did not cause clinical symptoms in mice, it caused significant damage to the gut, lungs, liver, kidneys, and brain. The emergence of this MRV strain may pose a threat to the health of animals and humans, and it is recommended that its epidemiology and recombination be closely monitored.

## Introduction

Mammalian orthoreoviruses (MRVs) are non-enveloped, double-stranded (ds) RNA viruses belonging to the genus Orthoreovirus within the family Reoviridae, which can cause symptomatic and asymptomatic infections in mammals. They have the potential to infect bats, pigs and most mammalian species, including humans (Tyler et al., 2001; Day, 2009). These viruses exhibit shared characteristics and represent the primary pathogens responsible for respiratory and gastrointestinal diseases, as well as encephalitis (Spriggs and Fields, 1982; Ren et al., 2022; Wang et al., 2023). The genome length of MRVs is approximately 23.5 kb. Each virus particle consists of 10 segments based of their electrophoretic mobilities: three large (L1, L2, and L3), three medium (M1, M2, and M3) and four small (S1, S2, S3, and S4) segments, which encode eight structural proteins (λ1, λ2, λ3, μ1, μ2, σ1, σ2, and σ3) as well as four nonstructural proteins (μNS, μNSC, σNS, and σ1s) (Kobayashi, 2014). The S1 genes encode the σ1 protein, which is closely associated with its virulence and its cell attachment and receptor binding properties (Meyer et al., 1997). The MRVs are divided into 4 serotypes: type 1 (MRV1) Lang (T1L), type 2 (MRV2) Jones (T2J), type 3 (MRV3) Dearing (T3D) and a putative 4 (MRV4) Ndelle, based on their σ1 proteins (Attoui et al., 2001; Lelli et al., 2016).

MRVs were first discovered and isolated from humans in 1950 (Sabin, 1959), and their segmented genome structures facilitate a phenomenon known as re-assortment, whereby segments from different species can exchange and combine within the same host, leading to the emergence of novel strains and promoting their evolution. This process is particularly significant when two or more MRVs co-infect the same host, and it allows for genetic diversity and potential adaptations to occur. This has the capacity to influence the dynamics of MRV populations and the interactions of the viruses with their hosts, making them being extremely recombinant and adaptive (Wang et al., 2023). MRVs exhibit a vast geographic distribution, encompassing a broad range of mammalian hosts, including but not limited to humans, bats, domestic animals such as swine and bovine, as well as various wild mammals (Kohl et al., 2012; Lelli et al., 2013; Anbalagan et al., 2014; Qin et al., 2017; Besozzi et al., 2019; Zhang et al., 2021; Ren et al., 2022; Wang et al., 2023). In China, there have been numerous reports on MRV infections. In 2017, a recombinant MRV strain with an L3 segment was isolated from diarrheal piglets in Heilongjiang. In 2022, a bat MRV strain was isolated from Xinjiang, exhibiting recombination with genetic fragments from humans, deer, cattle, and civets (Ye et al., 2020; Yan et al., 2022). Despite this, not all types of MRVs induce severe diseases in mammals (Lelli et al., 2016; Yamamoto et al., 2020).

MRVs infections typically manifest as a spectrum of afflictions encompassing respiratory and enteric pathologies, with certain instances eliciting notable neurological manifestations. There are also some instances of infection which have been linked to the development of severe and clinically significant diseases (Chua et al., 2008; Ahasan et al., 2019). MRVs have been found in the United States in various hosts, including humans, pigs and cattle. In the context of human infections, MRVs have been linked to respiratory and enteritis diseases, while those infected have also been reported to exhibit meningitis. However, in infected animals, MRVs primarily manifest themselves as enteritis diseases (Tyler et al., 2004; Tai et al., 2005; Ouattara et al., 2011; Thimmasandra et al., 2015).

In this study, a strain of type 3 MRV was isolated from diarrheic calves at a cattle farm in Liuzhou City, Guangxi, China. The genomic characteristics and *in vitro* growth features of the virus were analyzed, and its pathogenicity was assessed by its infection in mice. This study marks the first isolation and identification of a novel recombinant strain of Mammalian Orthoreovirus Type 3 (MRV-GXLZ2301) from bovine fecal matter in Guangxi Province. It reveals the genomic characteristics and recombination origins of the virus and evaluates its pathogenicity and transmissibility in a murine model. This work not only enhances our comprehension of MRV’s cross-host transmission and adaptability but also provides crucial scientific insights for the future prevention and control of MRV dissemination, holding significant implications for public health.

## Materials and methods

### Sample collection and detection

In August 2022, a severe outbreak of calf diarrhea was reported at a cattle farm in Liuzhou City, Guangxi, China. The affected calves exhibited symptoms of diarrhea, depression and reduced appetite. Samples of the diarrheal feces from the affected calves were collected by the Liuzhou City Animal Disease Prevention and Control Center. These samples were subsequently submitted to the Laboratory of Animal Infectious Diseases and Molecular Immunology at the College of Animal Science and Technology, Guangxi University, for testing and analysis. The samples were diluted with Dulbecco’s phosphate-buffered saline (DPBS) supplemented with an antibiotic/antimycotic solution. After dilution, the samples underwent three cycles of freezing and thawing, followed by centrifugation at × 10000 g at 4°C for 10 min. Subsequently, 1 mL aliquots of the fecal supernatants were collected and used for total RNA extraction as described previously (Luo et al., 2023).

Pan-viral family/genus PCRs and sequencing were performed for the following viral families: Coronaviridae, Herpesviridae, Picornaviridae and Reoviridae(The primers are as shown in Table 1) (Stanic, 1963; Li et al., 2017; Kia et al., 2021; Luo et al., 2023). The PCR was performed in a 50 μL reaction mixture containing 6 μL of template cDNA, 25 μL of Prime star Max DNA Polymerase (Takara Bio Inc, JPN), 1 μL of each forward and reverse primers and ddH2O was added to reach a total volume of 50 μL. PCR was conducted under the following conditions: an initial denaturation step at 95°C for 2 min, followed by denaturation at 95°C for 15 s, annealing at 55°C for 15 s, and extension at 72°C for 30 s, with a final extension at 72°C for 10 min. Then, the PCR products were purified by using an E.A.N.A. Gel Extraction Kit (Tiangen Inc, China) and directly sequenced by the method of Sanger (Sangon Bio Inc, Guangzhou, China). The purified PCR products were sequenced from both the 5′-3′ and 3′-5′ directions in order to ensure the absence of mutations in these segments. Each segment was sequenced at least twice.

**TABLE 1:** The primers used for detecting different viruses.

Primer name	Primes sequences (5′-3′)	Length of PCR product (bp)
BVDV-F	ATGCCCTTAGTAGGACTAGCA	292
BVDV-R	CAACTCCATGTGCCATGTACAGCAG	
MRV-5	GCATCCATTGTAAATGACGAGTCTG	416
MRV-6	CTTGAGATTAGCTCTAGCATCTTCTG	
MRV-7	GCTAGGCCGATATCGGGAATGCAG	344
MRV-8	GTCTCACTATTCACCTTACCAGCAG	
BCOV-F	ATGTCTTTTACTCCTGGTAAGCAATC	774
BCOV-R	ATGTCTTTTACTCCTGGTAAGCAATC	
BEV-F	CCGACTCCGCACCGATACGTCG	236
BEV-R	CTCTCAGAGCTACCACTGGGGT	
BPV-F	GCGAAAACACGACTTTG	554
BPV-R	GAGCCGTGTCACCAGTGTTA	
BHV-F	GCACACGACGGACGATGTG	126
BHV-R	GAGAGCGCGAACGAGTCGTAC	

### Cell culture

Madin-Darby bovine kidney (MDBK), Porcine Kidney-15 (PK-15), Verda reno (VERO), Baby Hamster Kidney-21 (BHK-21), Human Non-Small Cell Lung Cancer (A549), MARC-145 African Green Monkey Embryonic Kidney (MARC-145), and Madin-Darby canine kidney (MDCK) cells were cultured in Dulbecco’s modified Eagle’s medium (DMEM; Gibco Inc., USA) supplemented with 10% fetal bovine serum (FBS; Gibco Inc, USA) at 37°C in a humidified incubator with 5% CO2.

### Virus isolation and purification

The supernatant of positive fecal samples was first filtered through a 0.22-micron Millipore filter (Millipore Inc, USA), and then 10 micrograms per milliliter of N-tosyl-L-phenylalanine chloromethyl ketone (TPCK) trypsin (T1426, Sigma-Aldrich Inc, USA) was added to the MDBK cells. The cells were cultured in a 5% CO2 environment at 37°C for 3 days, after which the cell supernatant was collected. After three blind passages, the cell supernatant showing cytopathic effects (CPEs) was collected and used for total RNA extraction and RT-PCR detection. This isolation experiment was repeated three times to avoid contamination or other issues in the experiment.

The isolated virus was purified by performing plaque assays. Briefly, MDBK cells were seeded in 6-well plates and grows to nearly 90% confluence, and then the cells were inoculated with serially 10-fold diluted viral samples and incubated at 37°C for a further hour. The cells were then overlaid with a mixture of 2 × DMEM containing 1% low-melting agarose (Cambrex Inc, USA) and 2% FBS, followed by incubation for 3 days at 37°C in a 5% CO2 atmosphere. Subsequently, the media were carefully removed from the plates, and the cells were stained with 3–4 mL of staining solution, consisting of 0.5% crystal violet and 25% formaldehyde solution, for 15 min. To isolate a single viral clone, three viral plaques in the agarose were selected using pipettes, dispersed in DMEM, and then centrifuged. The resulting supernatants were used to infect MDBK cells, and the harvested viruses were serially passaged in MDBK cells. The entire experimental process was repeated three times to obtain the purified virus.

### NGS and analysis

Nucleic acid (NA) samples were subjected to reverse transcription using a primer that consists of a known sequence along with a random nanomer. Subsequently, a second primer extension reaction was performed using the same primer (26). Following this, the resulting extended fragments were amplified by PCR, utilizing the known sequence of the extension primer. The PCR amplicons obtained from the pre-amplification step were then purified and fragmented. Subsequently, libraries with dual index barcoding were constructed from the fragmented PCR products. The Illumina sequencing on a MiSeq instrument was carried out as previously described (Stanic, 1963; Li et al., 2017; Kia et al., 2021; Luo et al., 2023). After this step, the reovirus reads were identified by SURPI and subsequently isolated analyzed. Both *de novo* and reference-based assemblies were performed using Geneious v11.1.4 software and the consensus sequences of all the genomic segments generated were used for phylogenetic analysis.

### Virus electron microscopy analysis and *in vitro* biological characteristics

MDBK cells were infected with the isolated viruses and harvested at 48 h post-infection. A sample of the supernatant containing viral particles was centrifuged at 1,663 g for 20 min, followed by concentration at 106,982 g for 90 min using a Beckman L8-70M ultracentrifuge in an SW50.1 rotor. For negative staining, a drop of the sample was applied onto a Formvar carbon-coated grid and stained with 3% phosphotungstic acid (pH 6.3) for approximately 30 s. Subsequently, the specimen was inactivated using ultraviolet irradiation in order to ensure the inactivation of any virus particles which were then visualized using negative-staining electron microscopy.

To characterize the replication dynamics of the isolated MRVs, PK-15, VERO, BHK-21, MDCK, A549, MARC-145, and MDBK cells grown in a 96-well plates were infected with 10-fold serially diluted virus samples. After 3–5 days, viral titers for each cell type were used to quantify the number of virus particles based on the respective dilution-induced CPEs. Subsequently, PK-15, VERO, BHK-21, MDCK, A549, MARC-145, and MDBK cells were seeded in 24-well plates and these were infected separately with the virus at a multiplicity of infection (MOI) of 0.01. Then the supernatants from each infected cell line were collected at indicated time points (12, 24, 36, 48, 60, and 72 h), serially diluted in 10-fold increments and these were used to infect wells at each dilution. After 72 h post-inoculation (hpi), CPEs on individual cells were observed and counted, and the virus titers (TCID50) were calculated according to the Reed-Muench method (Stanic, 1963) to describe their viral growth kinetics.

### Antibody and western blotting analysis

In order to produce a S4 protein-specific antibody, the S4 gene from MRV-GXLZ2301 was first amplified using RT-PCR and subsequently inserted into the pET-32a (+) expression vector (Novagen Inc, GER), to generate a recombinant plasmid, pET32a-S4. This plasmid was subsequently introduced into BL21 (DE3) competent Escherichia coli cells. Following this, the cells were induced using 0.1 mM IPTG for a duration of 6 h and the resultant recombinant protein was purified using a His binding kit (Novagen Inc, GER).

PcAbs targeting the MRV-S4 protein were generated by immunizing Kunming mice with the purified MRV-S4 protein. These pcAbs were subsequently purified using protein A affinity chromatography. Protein lysates were then separated on a 12% SDS-PAGE gel and transferred onto a polyvinylidene fluoride membrane (PVDF; Millipore Inc, USA). The PVDF membranes were blocked with 5% non-fat dry milk for 2 h at 37°C and then incubated with either monoclonal antibodies against the 6-His-tag or anti-MRV-S4 pcAb overnight at 4°C. After five washes with Tris-buffered saline Tween-20 (TBST), the membranes were incubated with an HRP-conjugated secondary antibody (goat anti-mouse IgG; H + L; 1:5,000; Abmart Inc, CHN) for 1 h at 37°C. Finally, after five additional washes with TBST, the membrane-bound proteins were visualized using an enhanced chemiluminescence detection system.

### Indirect immunofluorescence assay (IFA)

PK-15, VERO, BHK-21, MDCK, A549, MARC-145, and MDBK cells were infected with MRV-GXLZ2301 at MOI of 0.01. After 48 h post-infection (hpi), the inoculum was removed, and the cell monolayer was washed three times with phosphate-buffered saline (PBS). The cells were then fixed with ice-cold methanol at −20°C for 30 min. Subsequently, the cells were washed five times with PBS and incubated with primary antibody (anti-MRV-S4 pcAb) at a dilution of 1:200 at 37°C for 2 h. After that, the cells were washed five times with PBS and incubated with secondary antibody (goat anti-mouse IgG H&L Alexa Fluor^®^ 488; Proteintech Inc. CHN) at 37°C for 1 h. The cells were then washed five times with PBS and stained with DAPI (Solarbio Inc., China) for 5 min to visualize the nuclei. Finally, the cells were observed under a fluorescence microscope.

### Phylogenetic analysis

To evaluate the optimal analysis method, we developed additional machine learning (ML) models for phylogenetic tree analysis. The maximum likelihood (ML) method with the general time-reversible (GTR) model and gamma (G) distribution rate was used to construct the phylogenetic tree of the S1 sequence. This analysis was performed using the Iqtree 1.6.1.2 software,^1^ with a bootstrap value of 1000. The evolutionary tree data was processed using Figtree 1.4.4.^2^ Other sequences of Orthoreovirus were downloaded from the NCBI GenBank database.^3^ A phylogenetic tree with 1000 bootstrap replicates was constructed using the neighbor-joining (NJ) method with the p-distance model in MEGA.7.0 and was beautified using an online website^4^ (Stanic, 1963; Xie et al., 2023). The pairwise genetic distance heatmap was constructed using the SDTv1.3 software.

The putative recombination origins of 222 MRV genomic sequences (including the virus strains isolated in this study) were then investigated using the RDP5.3 software package. Seven different methods were employed, namely RDP, GENECONV, MaxChi, Boostscan, Chimera, SiScan, and 3Seq, with default parameters. A p-value of less than 10-6 was considered significant if it was satisfied by at least six of the algorithms. Recombination events detected in the MRV genome were further confirmed using the Simplot software (v3.5.1, JHK University, Baltimore, MD, USA) with default parameters.

### Pathogenicity studies in neonatal mice

All animal studies were conducted with the approval of the Guangxi University Animal Experimentation Ethics Review Committee (ethics number GXU-2023-0142). Pregnant Kunming female mice were bred in-house in our laboratory and housed in a HEPA-filtered level 2 biosafety facility. All the animals were clinically healthy as well as being serologically and virologically confirmed as negative for BEV, BPV and MRV. The animals were provided with *ad libitum* access to food and water and were housed in a temperature-controlled room maintained at 24 ± 0.5°C. Twenty three-day-old mice pups were randomly assigned into two groups, with each group consisting of one dam and ten pups. In the experimental groups, 3-day-old Kunming mice were intra-peritoneally injected with 50 μL of viruses at 10^7^ TCID_50_. The negative control group received an injection of DMEM. Daily monitoring was conducted in order to observe any clinical symptoms.

### Replication kinetics of the virus in Kunming mice

The mice were dissected, and tissue samples including those from the hearts, livers, spleens, lungs, kidneys, intestines and brains, were collected at 3, 7, and 14 days post-infection (dpi) for detection of the tissue viral load at different time points by using a RT-qPCR method established in this study. Briefly, a pair of specific primers (F: 5′-CGACGGACTGACAGTATCGG-3′; R: 5′-CAGTCAGCTGCCCATCAGAA-3′) were designed targeting the MRV S1 specific region. The RT-qPCR protocol consisted of an initial denaturation at 95°C for 30 s, followed by 40 cycles of denaturation at 95°C for 10 s and annealing at 64°C for 10 s. The melt curve acquisition program included steps at 95°C for 15 s, 60°C for 60 s, and a final cooling step at 37°C for 30 s.

### Histopathology of virus-infected Kunming mice

After a 3-day infection period, various tissues samples from the hearts, livers, spleens, lungs, kidneys, brains and intestines) were harvested from Kunming mice and fixed in a 4% (v/v) paraformaldehyde solution for histopathological analysis. The fixed tissues were then embedded in paraffin blocks and sectioned into 5 mm slices, which were subsequently mounted on glass slides. Microscopic examination of these sections was performed following haematoxylin-eosin (HE) staining to assess the extent of pathological damage in the tissues.

## Results

### Isolation and identification of a MRV strain

**FIGURE 1:**
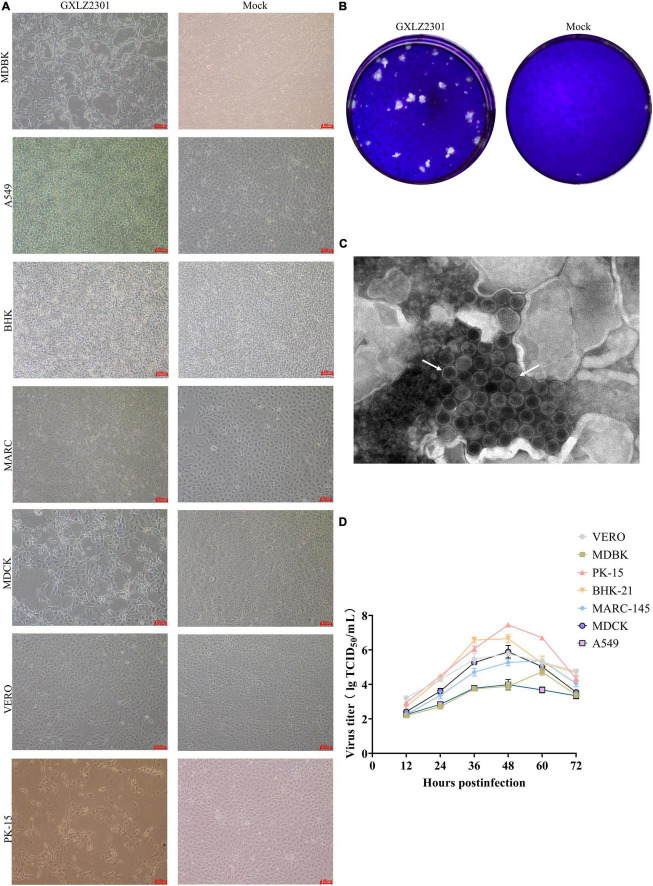
Growth characteristics and morphology of the novel isolated MRV strain. **(A)** CPEs in MDBK, PK-15, VERO, BHK-21, MDCK, A549 and MARC-145 cells infected with the GXLZ2301 strain. Mock- and virus-infected cells were observed at 36 hpi. **(B)** Virus particle morphology of the MRV GXLZ2301 strain using electron microscopy. **(C)** Plaque morphology of the MRV-GXLZ2301 strain on MDBK cells. **(D)** Growth kinetics of the isolated strain in MDBK, PK-15, VERO, BHK-21, MDCK, A549 and MARC-145 cells. At different times, post-infection, the cell supernatants were collected and the virus titers were determined as TCID_50_ values. The results represent the means of three independent experiments, the viral multi-step growth curve was plotted using GraphPad version 9.5.1.

Bovine diarrhoeic fecal specimens were subjected to RT-PCR analysis using specific primers to detect several major pathogens. The results indicated that these were positive for MRV, and negative for BVDV, MRV, BCoV, BEV, BPV and BHV. After inoculating MDBK cells with the positive fecal supernatants, typical CPEs were observed after blind passaging for two generations. The cells showed single-cell necrosis and syncytium formation, the cytoplasm became more granular, and the characteristics of CPE included rounding, shrinking, granulation, networking and separation, and vacuole formation. By 30–48 h, a significant number of infected cells were detached from the culture dish (Figure 1A). The isolated strain was purified using plaque assays in MDBK cells (Figure 1B), and the GXLZ2301strain was obtained. Icosahedral, non-enveloped, uniform-sized particles with a double-layered structure were observed under the transmission electron microscopy (TEM) (Figure 1C), and they showed a strong resemblance to members of the Reoviridae family.

Supernatants collected from the aforementioned pathological cells, when added to VERO, PK-15, BHK-21, MARC-145, and A549 cell lines, induced CPE. The multistep growth curves of the isolated strains were evaluated at MOI of 0.01 across different cell lines. Results indicated that the viral titer of GXLZ2301 was highest in PK-15 cells, reaching a peak titer of 10^7.46^ at 48 h. Similarly, MRV-GXLZ2301 achieved peak viral titers at 48 h in VERO, BHK-21, MDCK, and A549 cells, the viral titers were 10^5.78^ TCID50/0.1 mL, 10^6.65^ TCID50/0.1 mL, 10^5.89^ TCID50/0.1 mL, and 10^3.98^ TCID50/0.1 mL, respectively. However, the highest viral titers in MDBK and MARC-145 cells were reached at 60 h, being 10^4.73^ TCID50/0.1 mL and 10^5.37^ TCID50/0.1 mL, respectively (Figure 1D). All six tested cell types were susceptible to infection, demonstrating that GXLZ2301 possesses broad infectivity and cell tropism across various types of mammalian cells, including those of human origin.

**FIGURE 2:**
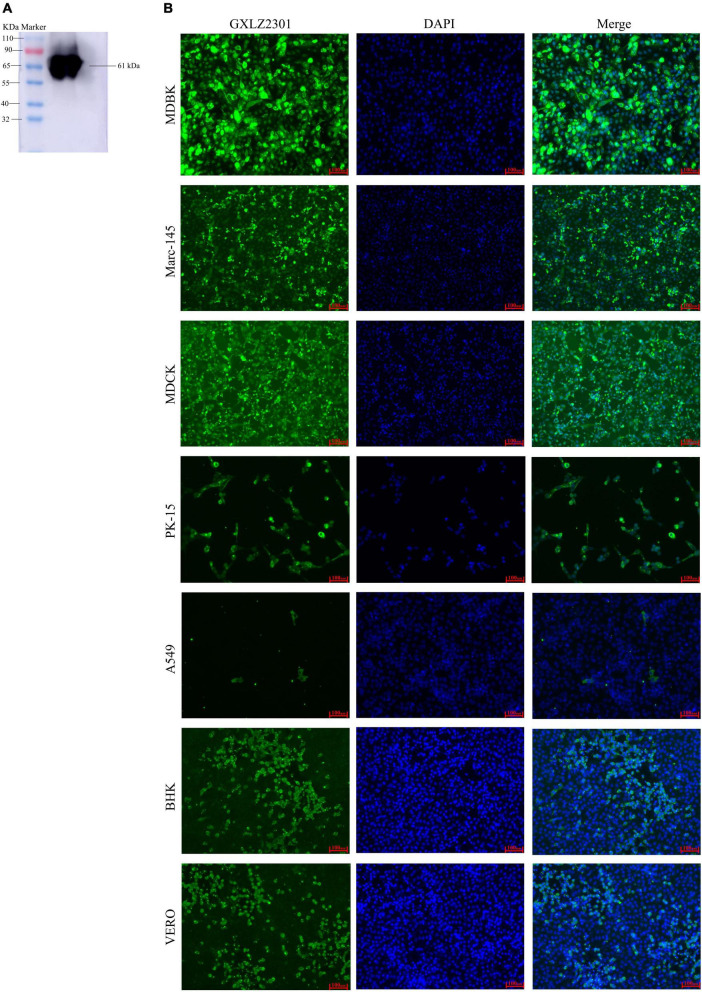
Analysis of the recombinant protein and IFA. **(A)** Western blot analysis of the expressed recombinant, MRV-GXLZ2301-S4, shows reactivity with the polyclonal antibodies prepared in this assay. **(B)** IFA analysis of the S4 protein expression was conducted in MDBK, PK-15, VERO, BHK-21, MDCK, A549 and MARC-145 cells. The virus-infected cells were fixed and stained using an anti-buffalo MRV-S4 PcAb and goat anti-mouse IgG H&L (green). The cell nuclei were visualized by DAPI (blue) staining.

To generate antibodies against the MRV S4 protein, the S4 gene of the GXLZ2301 strain was cloned into the pET-32a (+) expression vector and transformed into the Escherichia coli strain BL21 (DE3). Subsequently, a 61 kDa recombinant protein was successfully expressed and purified using a His-tag purification kit (Figure 2A). Kunming mice were immunized with recombinant proteins, and after three immunizations, blood samples were collected to obtain polyclonal antibodies (pcAbs) against the MRV S4 protein. These antibodies were then utilized for Western Blot (WB) and Immunofluorescence Assay (IFA) testing across various cell types, as shown in Figure 2B.

### Genotypic characterization and diversity of MRVs in Guangxi, China

The genome sequences of GXLZ2301 were obtained by next-generation sequencing. No additional viral sequences were found in the deep sequencing data, indicating the absence of contamination from other viruses. The substantial sequence identity of GXLZ2301 sequences further corroborated our findings from immunofluorescence and electron microscopic analyses. The total length of the MRV genome was detrmined to be 23567 nucleotides (nt). The 5′ and 3′ untranslated regions (UTRs) ranged in length from 12 to 31 and 32 to 79 nt, respectively, with variations from the prototype MRV3-T3D (Table 2).

**TABLE 2:** MRV-GXLZ2301 bovine orthoreovirus strain show altered UTRs.

Segment	Size (bp)	5′end		ORF/protein				3′end	
		Terminal sequence	UTR (bp)	Region	Size (aa)	Class	Protein function	UTR (bp)	Terminal sequence^a^
L1	3,854	**GCU***C*UU	18	19-3822	1,267	λ3	RNA-dependent RNA polymerase	32	ACU**CAUC**
L2	3,912	**GCUA**UU	12	13-3876	1,288	λ2	Guanyltransferase, methyltransferase	33	AUU**CAUC**
L3	3,901	**GCUA**AU	13	14-3841	1,275	λ1	RNA binding, NTPase, helicase, RNA triphosphatase	60	ACU**CAUC**
M1	2,303	**GCUA**UU	13	14-2224	736	μ2	Binds RNA NTPase	79	CUU**CAUC**
M2	2,203	**GCUA**AU	29	30-2156	708	μ1	Cell penetration, transcriptase activation	47	GAG**G***G***UC**
M3	2,244	**GCUA**AA	18	19-2184	721	μNS	Unknown	60	AUU**CAUC**
S1	1,426	**GCUA**UU	12	13-1380 71-432	455 120	σ1 σ1s	Cell attachment	46	GAG**C***G***UC**
S2	1,331	**GCUA**AU	18	19-1275	418	σ2	Binds dsRNA	56	AUU**CAUC**
S3	1,198	**GCUA**AA	27	28-1128	366	σNS	Inclusion formation, binds ssRNA	70	AAU**CAUC**
S4	1,195	**G**G**U***G*UG	31	32-1129	365	σ3, σ3a, σ3b	Binds dsRNA	66	AUU**CAUC**

^a^The 5 and 3 untranslated regions (UTRs) of GXLZ2301 show mutations on the L1, M2, S1, and S4 segments. The conserved terminal sequences are shown in boldface, and mutations are italicized.

The results analysis showing the highest nucleotide and amino acid identities for each genome segment against the publicly available sequences from the GenBank are reported in Table 3. Sequence analysis showed that the S1 segment displayed 93.91 % sequence identity with a bat-derived MRV3 (T3/Tadarida_teniotis-Italy-2013) and the L1 segment was closely related to the bovine MRV00304-USA-2014 MRV3 strain (86.68 % homology). The remaining L and M segments were found to be highly homologous to human-derived MRV2 and the S2-4 segments were highly homologous to pig-derived MRVs.

**TABLE 3:** The highest nucleotide identities of MRV strains with each gene segment of the novel reassortant MRV-GXLZ2301.

Genetic segment	Identity (%)		MRV strain	Serotype	Host	GenBank no.
	nt	aa				
L1	86.68	96.68	MRV00304-USA-2014	3	Bovine	KJ676379
L2	81.98	94.64	SI-MRV08-Slovenia-2011	2	Human	MT518185
L3	83.50	97.60	Jones L3-USA-1999	2	Human	AF129821
M1	79.72	90.08	Reovirus 2 minor reovirus-USA-1999	2	Human	AF124519
M2	82.47	96.05	SI-MRV08-Slovenia-2011	2	Human	MT518188
M3	81.31	90.97	SI-MRV08-Slovenia-2011	2	Human	MT518189
S1	93.91	91.21	T3/Tadarida_teniotis-Italy-2013	3	Bat	JQ979276
S2	85.20	97.13	BM-100-USA-2014	3	Porcine	KM820751
S3	90.57	98.36	FS-03-USA-2014	3	Porcine	KM820762
S4	91.08	96.71	sR1590-China Taiwan-2019	2	Porcine	LC482247

**FIGURE 3:**
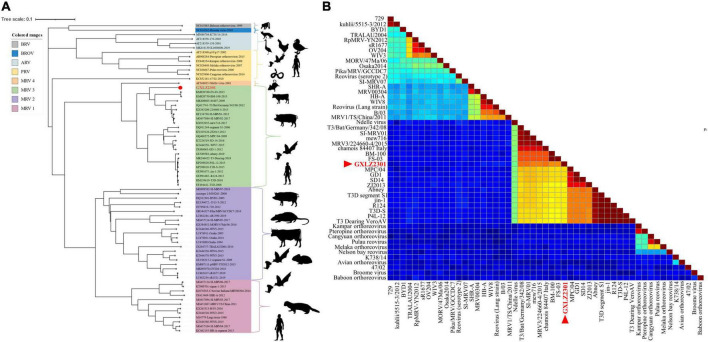
Phylogenetic analysis of GXLZ2301. **(A)** Phylogenetic tree of the S1 segment of novel reassortant MRV-GXLZ2301. The related MRV strains were downloaded from the GenBank, and the open reading frame of each gene segment was used for building the phylogenetic trees. The MRV isolate identified in this study is labeled with a round red dot. This analysis was performed using the Iqtree 1.6.1.2 software (http://www.iqtree.org/), with a bootstrap value of 1000. **(B)** Based on the GXLZ2301 S1 segment and paired genetic distance heatmap of other MRV3 strains, the red font denotes GXLZ2301. The pairwise genetic distance heatmap was built using SDTv1.3 software.

**FIGURE 4:**
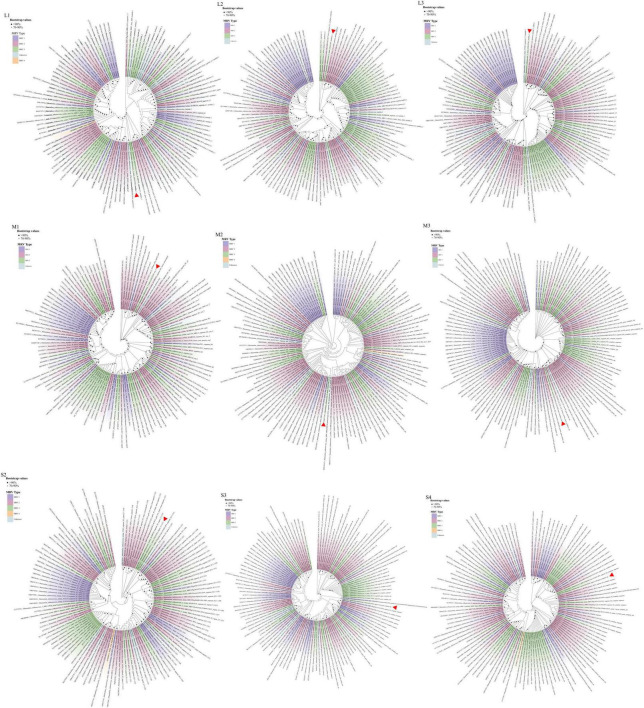
The phylogenetic analysis of GXLZ2301, based on the complete nucleotide coding sequences of fragments S2-S4, M1-M3, and L1-L3. Different MRV lineages are denoted by distinct color blocks. The red arrow indicates GXLZ2301. The phylogenetic tree was constructed using the Neighbor-Joining (NJ) method in MEGA.7 and beautified online at Chiplot (https://www.chiplot.online/).

Phylogenetic analysis of the GXLZ2301 isolate reveals an evolutionary relationship completely independent of other MRVs. The S1 phylogeny indicates that GXLZ2301 belongs to the serotype 3 (MRV3) branch, with MRV3 being identified in cattle for the first time, the GXLZ2301 strain’s S1 gene segment is on the same evolutionary branch as the U.S. strains FS-03 and BM100, which were reported in 2014 (Figure 3A). On the other hand, a heatmap of pairwise genetic distances based on the S1 segment shows low sequence similarity between GXLZ2301 and other MRV3 strains (Figure 3B). Notably, the L1 segment of the GXLZ2301 strain shares the same evolutionary branch with MRV00304, which was reported in 2014; the L2, L3, M1, and M3 segments are on the same evolutionary branch as the classic type 2 MRV Jones reported in the United States and SI-MRV08 reported from Slovenia in 2011; the M2 and S3 segments are on the same evolutionary branch as the Italian strain 18RS29002 reported in 2020; the S2 segment is evolutionarily similar to the S1 segment, existing on a separate branch without any strains clustering with it; the S4 segment is on the same evolutionary branch as the Japanese strain Ishi-Ueno-10, which was reported in 2021 (Figure 4). Although clustering with the MRV3 GXLZ2301 group in the S1 phylogenetic tree, it forms a distinct branch (Figure 3A). In contrast, in the phylogenetic trees based on the other nine gene segments, it aligns with other MRV subtypes on the same branch. These findings suggest that GXLZ2301 may represent a new lineage of mammalian orthoreovirus.

### Recombination analysis of GXLZ2301 to explore the potential evolutionary process for MRVs

**FIGURE 5:**
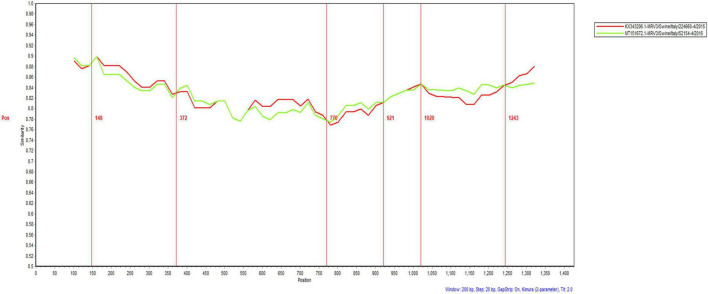
Recombination analysis of the S1 protein of the isolated MRV-GXLZ2301 strain. Recombination analysis was performed by using the Simplot software package.

An analysis of recombination events in ten segments of isolated MRVs was performed using the RDP5 and Simplot software packages (Figure 5 and Table 4). The results indicated that a genetic recombination event occurred within the isolated MRV-S1 segment. The GXLZ2301 strain was a recombinant from the MRV type 3 strain 52154-4 (MT151672, Italy, 2016) and the 224660-4 strain (KX343206, Italy, 2015). A similarity plot analysis showed that the genome had six recombination breakpoints (positions of alignment) that were located in σ1s protein (nt 148 and nt 372) and σ1 protein (nt 770, nt 921, nt1020 and nt 1243). No recombination events were found in the remaining segments after extensive software analysis.

**TABLE 4:** Information on recombination events of the MRV-GXLZ2301 isolate as detected by RDP5 software.

Strains	Parental sequence		Detection methods (*p*-value)						
	Minor	Major	RDP	GENE CONV	Bootscan	Maxchi	Chimera	SiScan	3Seq
MRV-GXLZ2301-S1	52154-4 (MT151672)	224660-4 (KX343206)	3.062 × 10^−4^	1.370 × 10^−7^	2.062 × 10^−8^	9.616 × 10^−5^	1.929 × 10^−11^	4.088 × 10^−7^	5.086 × 10^−2^

### Pathogenicity and transmissibility of GXLZ2301 in mice

**FIGURE 6:**
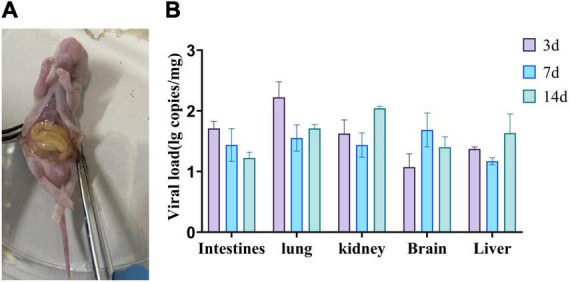
Pathogenicity of bovine orthoreovirus isolates. **(A)** Anatomy of a representative infected mouse. **(B)** Replication kinetics of the MRV-GXLZ2301 strain in infected mice. 3-day-old Kunming mice were intra-peritoneally injected with 107 TCID50 of the MRV-GXLZ2301 strain and three mice were dissected at 3, 7 and 14 dpi, respectively. Their livers, lungs, kidneys, brains and intestines were used for virus detection by RT-qPCR. The data are expressed as means ± SDs of three samples. The error bars represent the SD from the triplicate within each experiment.

The newly identified MRV GXLZ2301 was intra-peritoneally injected into 3-day-old healthy Kunming mice in order to assess its pathogenicity. The results revealed that the mice in the virus-inoculated group exhibited symptoms of depression and reduced weight gain. After euthanizing the infected mice, postmortem examinations revealed increased abdominal fluid and deep yellow coloration in the intestines as well as bleeding from the hearts and lungs (Figure 6A).

To further explore the distribution of the viral antigens in the mice, we collected different tissue samples from the mice on days 3, 7, and 14 for viral RNA detection. Virus RNA was found in the intestines, lungs, livers, brains and kidneys of infected mice, and the viral load in each organ varied with time, as shown in Figure 6B. The intestines and lungs showed the highest viral loads on day 3, while the livers, brains and kidneys reached their peak viral loads on days 7 or 14, suggesting that the virus initially infected the intestines and lungs, and then gradually spread to the rest of the tissues. These findings indicated that GXLZ2301 was capable of effective replication in Kunming mice.

**FIGURE 7:**
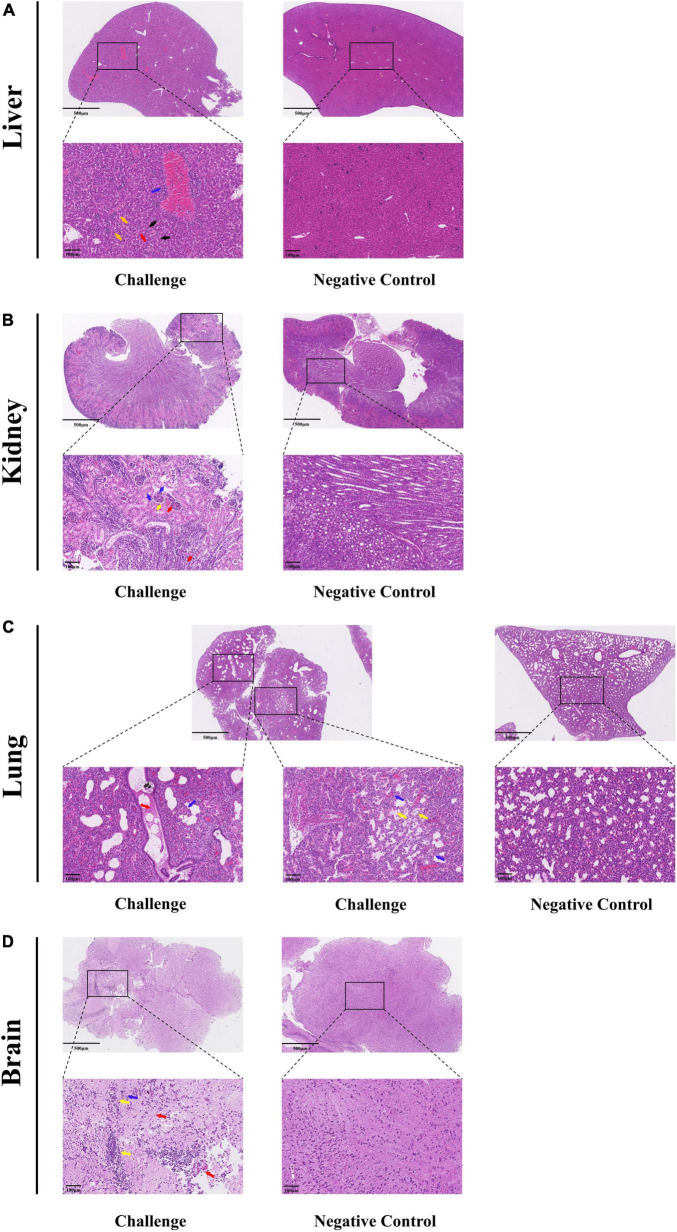
Histopathological analysis of MRV-GXLZ2301-infected Kunming mice 3 days after infection. Livers **(A)**, kidneys **(B)**, lungs **(C)** and brains **(D)** of the MRV-GXLZ2301 infected mice were obtained. At the indicated time points post-infection, the paraffin sections of tissues were stained with haematoxylin–eosin (HE) and observed under a light microscope. All observations were performed with 200× magnification.

Under optical microscopy, HE-stained histopathological sections were used to assess the tissue damage. The results of RT-qPCR revealed consistent pathological changes in the livers, kidneys, brains and lungs of Kunming mice. In the liver, there were inflammatory cell infiltrations around vessels (blue arrows) as well as variable-sized aggregates (yellow arrows). The hepatocytes showed diffuse vacuolar degeneration and nuclear pyknosis (black arrows) and the liver sinusoids were congested, with increased white blood cells (red arrows) and hepatic cords disorganization (Figure 7A). The kidneys exhibited cortex integrity, although some tubular cell necrosis (blue arrows) was observed with minimal glomerular serous exudates (yellow arrows). Interstitial congestion and hemorrhage were present (red arrows) in the cortex and medulla (Figure 7B). The lungs had congested alveolar walls, inflammatory cell infiltration, alveolar septa widening (blue arrows), and some alveoli with serous exudates (yellow arrows). The bronchi showed some serous materials as well as red blood and inflammatory cells (red arrows) (Figure 7C). The brain tissue showed neuronal necrosis (blue arrows), increased inflammatory cells with nodules (yellow arrows) as well as mild congestion and hemorrhage (red arrows) (Figure 7D).

## Discussion

MRVs can infect many mammalian species, including cattle, sheep, horses, pigs, dogs, cats, mice and humans, causing respiratory, gastrointestinal, reproductive as well as neurological diseases in these hosts (Li et al., 2015; Gatherer et al., 2021). Due to the apparent lack of species barriers, MRVs have the potential to cross from animals to humans, making it an important zoonotic pathogen (Li et al., 2015). The prevalence and isolation of MRV have been reported worldwide. For instance, MRVs have been isolated from a 6 and a half weeks old child with meningitis as well as in a case of acute necrotizing encephalopathy in Europe (Tyler et al., 2004; Ouattara et al., 2011). In the United States, the sero-positivity rate for MRVs in pigs is 52% (Wang et al., 2021), while it is 19% in South Korea (Kwon et al., 2012). In China, MRV has also been isolated from masked palm civets and plateau pikas. (Li et al., 2015; Zong et al., 2023). However, to date, there have been no reports of cattle-associated MRVs in Guangxi, China.

In this study, an MRV strain from the feces of a diarrheic cattle herd in Liuzhou, Guangxi was successfully isolated in MDBK cells and the virus replication characteristics in different cell types including PK-15, VERO, BHK-21, MDCK, A549 and MARC-145 cells were investigated. Surprisingly, the MRV GXLZ2301 strain replicated most efficiently in PK-15 cells, while its replication titer was relatively lower in VERO cells. This was different from previous reports whereby VERO cells were found to be the best cells for MRV infectivity (Singh et al., 2022). We speculated that this phenomenon might be correlated with the cellular infectivity imposed by different infective viral strains, a phenomenon observed in other viruses (Matoba et al., 1993; Bussiere and Miller, 2021). Interestingly, we observed that the isolated virus reached its highest titer in MDBK and MARC-145 cells at 60 h, whereas in other cell types reached their peak titers were at 48 h. This suggested a possible unique replication mechanism for this MRV in MDBK cells. Infection and robust replication within the PK-15 cell line highlights its propensity for optimal propagation. Based on the reports of the infectivity of type 3 MRVs in pigs (Thimmasandra et al., 2015; Lelli et al., 2016; Singh et al., 2022), this suggested to us that this virus could potentially pose major economic concerns for the swine industry. These findings underscore the significance of further exploration of MRVs in our future investigations.

Based on the phylogenetic analysis of the GXLZ2301 S1 segment, although it forms an independent branch, it is classified as type 3 MRV. In Hrdy et al. (1979) successfully isolated type 3 MRV from cattle and studied the migration patterns of its genomic segments (Hrdy et al., 1979). ubsequent reports on type 3 MRV in cattle have remained elusive. Interestingly, the analysis of its S1 gene segment shows that it has an independent clustering, which may suggest the existence of undiscovered subvariants within this group type; the remaining segments in the phylogenetic tree are on the same branch as types 1 and 2 MRV, which is different from the type 3 MRV isolated from plateau pikas reported in China in 2023 (Zong et al., 2023). This suggests that this strain has been circulating and evolving in Guangxi for a considerable period, leading to the emergence of unique segment recombination. Furthermore, the typing of MRV currently mainly relies on the S1 segment for confirmation, and we will also further confirm whether it belongs to a completely new subtype. Homology analysis of the various segments indicates that GXLZ2301 may have become a new type of MRV virus through gene segment recombination during mixed infections involving multiple human, swine, and bovine MRV isolates in a natural environment. However, we have not yet simulated the growth characteristics of this virus in the intestine, so the growth mechanism of GXLZ2301 in the intestine remains unknown.

Recombination events in MRVs have been widely reported in the literature, but there is limited research on the analysis of these (Decaro et al., 2005; Kia et al., 2021; Ren et al., 2022; Singh et al., 2022; Wang et al., 2023). In this study, RDP5 and Simplot software packages were used to assess potential recombination events involving GXLZ2301. The results suggest that the S1 segment might have undergone recombination with a swine-derived MRV strain reported in Italy during 2015-2016, although no recombination events were detected in other segments after software analysis. The S1 segment constitutes a crucial viral subtype and protein functional fragment within the virus. The S1 segment of the GXLZ2301 strain is derived from the recombination of two porcine MRVs, and its favorable growth characteristics in porcine PK-15 cells corroborate our findings. However, the precise mechanisms underlying its replication remain to be further investigated. This virus strain exhibits recombination characteristics of human, bovine, and porcine genomic fragments, indicating that it may have acquired new genetic material through co-infection in a common host, thereby enhancing its genetic diversity and adaptability. This recombination event may provide the virus with new biological properties, such as altered cell tropism or increased pathogenicity (Ichikawa et al., 2023; Puente et al., 2023). From an epidemiological perspective, the isolation of GXLZ2301 suggests that MRV may be more widely present in animal populations and has the potential to generate new epidemic strains through recombination (Mao et al., 2024; Zhou et al., 2024).

The orchestration of Reovirus attachment and internalization is known to rely on the intricate interplay between three distinct viral capsid proteins and a multitude of host factors (Koehler et al., 2019, 2021). The Reovirus σ1 protein serves as an attachment factor, engaging with sialic acid (SA) with modest affinity (Barton et al., 2001a; Stencel-Baerenwald et al., 2014). Furthermore, the same σ1 protein can establish a notably stronger binding affinity with the junctional adhesion molecule A (JAM-A) at a distinct protein interface (Barton et al., 2001b; Guglielmi et al., 2007; Reiter et al., 2011). While the protein functions of various segments have been predicted, apart from the S1 segment, there is a scarcity of research reports on the other segments (Shang et al., 2023). Understanding the potential functions of these genes may be crucial in understanding the pathogenic mechanisms of these viruses. We recommend that further detailed studies on these segments for future clarification.

Traditionally, MRVs have been considered a pathogen that cause mild respiratory and gastrointestinal infections with no significant clinical impact (Wang et al., 2015). However, recent studies have shown that MRVs can lead to severe diseases, causing upper respiratory tract infections, diarrhea, encephalitis and fatalities in both humans and mammals. In fact, the pathogenesis of MRV infections has been extensively studied in mammals and adult animals, where these have lead to systemic viral replication, illness and even death. MRV strains exhibited serotype-specific variations in cell and tissue tropism as well as viral dissemination mechanisms (Tardieu and Weiner, 1982; Dichter and Weiner, 1984; Tyler et al., 1986). In this study, we infected Kunming mice with the isolated virus by intraperitoneal injections. The results confirmed that GXLZ2301 is pathogenic to 3-day-old Kunming mice, although they did not display clear clinical symptoms, they did exhibit features such as behavioral lethargy and decreased growth rates and necropsy revealed ascites accumulation. In addition, RT-qPCR detected the virus in the intestines, lungs, kidneys, brains and livers. HE staining of tissue slices showed lesions in the lungs, kidneys, brains and livers. Further research is needed to understand the pathogenicity of GXLZ2301 in other animals.

MRV may infect unhealthy animals, enhance the pathogenicity of other viruses, and lead to increased host mortality rates. Previous studies have shown that MRV infection may act synergistically with other pathogens, exacerbating the process of co-infections. For instance, dogs co-infected with MRV and CPV-2 (Canine Parvovirus 2) may die from severe enteritis (Decaro et al., 2005). Considering that the isolated MRV shares the highest similarity with human and swine MRV strains, they may pose a significant threat to animal breeding. Research on the novel swine-associated recombinant MRV-GXLZ2301 suggests that future research directions should include in-depth exploration of the virus’s genetic evolution, cross-host transmission mechanisms, pathogenicity and pathogenesis, as well as host immune responses (Li et al., 2024; Shang et al., 2024; Welsh et al., 2024). Additionally, there should be an enhanced epidemiological investigation and environmental monitoring of MRV to detect new epidemic strains early on. In terms of monitoring and control strategies, it is recommended to establish national and international monitoring networks, improve diagnostic capabilities, implement risk assessment and early warning systems, strengthen biosafety measures, conduct education and training, formulate outbreak response plans, and enhance international cooperation and information sharing. These measures will provide a scientific basis for the prevention and control of MRV, reducing its impact on public health and animal health.

## Conclusion

In summary, we successfully isolated a MRV strain from diarrheal fecal in cattle being farmed in Guangxi, China. By sequencing its complete genome and studying its growth characteristics in cell cultures, we revealed its molecular and virological features and this has provided a foundation for understanding the genetic relationships among Reoviruses. Pathogenicity experiments showed that the newly isolated GXLZ2301 strain could infect mice via intraperitoneal injections, causing lesions in the intestines, lungs, liver, kidneys and brain. Although no significant clinical symptoms were observed, the mice did exhibit slow weight gain and lethargy. Based on these findings, we recommend closely monitoring the epidemiology of MRVs and conducting surveillance for the possibility of emerging new strains. Additionally, it is advisable to develop candidate vaccines for MRVs to be prepared for potential future outbreaks. Such efforts will contribute to the prevention and control of MRV transmission and pathogenicity in the future.

## Data availability statement

The datasets presented in this study can be found in online repositories. The names of the repository/repositories and accession number(s) can be found in this article/supplementary material.

## Ethics statement

The animal study was approved by the Guangxi University Animal Experimentation Ethics Review Committee. The study was conducted in accordance with the local legislation and institutional requirements.

## Author contributions

YL: Investigation, Methodology, Writing – original draft. YW: Investigation, Methodology, Software, Writing – original draft. WT: Methodology, Investigation, Writing – original draft. CW: Resources, Writing – original draft. HL: Methodology, Writing – original draft. XW: Visualization, Writing – review and editing. JX: Visualization, Writing – review and editing. JW: Methodology, Writing – original draft. KO: Writing – review and editing. YC: Writing – review and editing. ZW: Writing – review and editing. YQ: Writing – review and editing. YP: Funding acquisition, Writing – review and editing. WH: Writing – review and editing.

## Footnotes

1
http://www.iqtree.org/

2
http://tree.bio.ed.ac.uk/software/figtree/

3
https://www.ncbi.nlm.nih.gov/

4
https://www.chiplot.online/
